# The Cutinase Bdo_10846 Play an Important Role in the Virulence of *Botryosphaeria dothidea* and in Inducing the Wart Symptom on Apple Plant

**DOI:** 10.3390/ijms22041910

**Published:** 2021-02-14

**Authors:** Bao-Zhu Dong, Xiao-Qiong Zhu, Jun Fan, Li-Yun Guo

**Affiliations:** College of Plant Protection and Key Lab of Pest Monitoring and Green Management, MOA, China Agricultural University, Beijing 100193, China; dongbaozhu2020@imau.edu.cn (B.-Z.D.); mycolozhu@cau.edu.cn (X.-Q.Z.); jfan@cau.edu.cn (J.F.)

**Keywords:** *Botryosphaeria dothidea*, cutinase, pathogenic mechanism, wart formation, defense response

## Abstract

*Botryosphaeria dothidea* is a pathogen with worldwide distribution, infecting hundreds of species of economically important woody plants. It infects and causes various symptoms on apple plants, including wart and canker on branches, twigs, and stems. However, the mechanism of warts formation is unclear. In this study, we investigated the mechanism of wart formation by observing the transection ultrastructure of the inoculated cortical tissues at various time points of the infection process and detecting the expression of genes related to the pathogen pathogenicity and plant defense response. Results revealed that wart induced by *B. dothidea* consisted of proliferous of phelloderm cells, the newly formed secondary phellem, and the suberized phelloderm cells surrounding the invading mycelia. The qRT-PCR analysis revealed the significant upregulation of apple pathogenesis-related and suberification-related genes and a pathogen cutinase gene *Bdo_10846*. The *Bdo_10846* knockout transformants showed reduced cutinase activity and decreased virulence. Transient expression of Bdo_10846 in *Nicotiana benthamiana* induced ROS burst, callose formation, the resistance of *N. benthamiana* to *Botrytis cinerea*, and significant upregulation of the plant pathogenesis-related and suberification-related genes. Additionally, the enzyme activity is essential for the induction. Virus-induced gene silencing demonstrated that the *NbBAK1* and *NbSOBIR1* expression were required for the Bdo_10846 induced defense response in *N. benthamiana*. These results revealed the mechanism of wart formation induced by *B. dothidea* invasion and the important roles of the cutinase Bdo_10846 in pathogen virulence and in inducing plant immunity.

## 1. Introduction

*Botryosphaeria dothidea* is a pathogen with worldwide distribution, infecting hundreds of species of economically important woody plants, including apple, pear, grape, blueberry, poplar, and eucalyptus [1]. It causes fruit rot, wart, and canker on twigs, branches, and stems, dieback of twigs and branches, or plant death, and is a constant threat to agriculture, commercial forest industry, and native ecosystems [1,2,3,4,5]. The apple ring rot (white rot) caused by *B. dothidea* is one of the most destructive diseases in the major apple production region in China [6]. Wart, canker, and dieback are the characteristic symptoms caused by this pathogen on apple twigs, branches, and stems (Figure S1A) [6]. However, the mechanism of wart formation and the molecular mechanism of the pathogenicity of this important pathogen is unclear.

During evolution, plants have developed physical barriers to protect plants from the adverse environment and the attack from other organisms. Cuticle, which consists of cutin, serves as an epidermal barrier coating the outer surface of the plants [7,8]. The phellem layer in the cortex of root and stem, which consists of suberin, serves as a physical barrier at the interface between the secondary development tissue and the environment [9]. Both cutin and suberin are polyesters, although their monomers differ in the length of fatty acid chains [9]. For example, in *Arabidopsis thaliana*, the suberin monomers consist of C16-C26 fatty acids, whereas the cutin monomers consist of C16-C18 fatty acids [10,11,12]. When the cuticle is damaged by pathogen invasion, genes of plant suberification-related proteins, such as Myb transcriptional factors, CYP450, and feruloyl-CoA-acyl transferase (FCoAT), will be activated to repair the defects through generating phellem layer [13,14,15,16,17]. Therefore, both cuticle and phellem layers are important physical barriers for plants against pathogen invasions.

Cutinase is a serine esterase, which belongs to the α/β hydrolase superfamily, and contains a classical Ser–His–Asp (S–H–D) catalytic triad and a Gly–Tyr–Ser–Gln–Gly (GYSQG) conserved catalytic site [18,19]. Cutinase can hydrolyze natural polyester (such as cutin and suberin), insoluble triacylglycerols, and low-molecular-weight esters [18,20,21,22]. Studies have shown that plant fungal pathogens generally can produce cutinase to hydrolyze the cuticle and phellem layer [23,24]. Moreover, cutinases of many plant pathogens are virulence-associated. In *Moniliania fructicola,* the overexpression of the cutinase gene *MfCUT1* promotes the pathogen virulence on *Prunus* spp. [25]. In *Curvularia lunata*, deleting the *ClCUT7* decreases the pathogen virulence on maize leaves [26]. Additionally, cutinase plays an important role in forming the infection structure of plant fungal pathogens [27]. For example, the *MoCut2* of *Magnaporthe oryzae* and the *CtCUT1* of *Colletotrichum truncatum* are required for the appressorium formation of these pathogens [27,28].

In addition to the physical barriers, plants have developed a sophisticated defense system to counter microbial invasions. Upon infection, plant pattern recognition receptors (PRRs), pathogen-associated molecular patterns (PAMPs), or damage-associated molecular patterns (DAMPs) activate pattern-triggered immunity (PTI) responses [29]. PAMPs are conserved molecules present in microbes but absent in host plants [30,31,32]. DAMPs are plant molecules released after plant tissue or cell damage [33]. Recently, studies have revealed that some cutinases of plant pathogen can induce plant immunity. The *A. thaliana* expressing the cutinase gene (AAA33334.1) of *Fusarium solani* is immune to *Botrytis cinerea* [34]. The cutinase VDCUT11 of *Verticillium dahlia* plays an important role in the interaction between plants and pathogens in the apoplast region at PAMPs triggered immunity (PTI) [35].

Interestingly, the recently published genome data of several *B. dothidea* isolates revealed many cutinase genes in this pathogen [36,37]. Ding has found 13 cutinase genes (Table S1) in *B. dothidea* and four of which are upregulated in the process of infecting apple shoots [38]. However, their roles in the infection process are unknown, mainly due to the lack of gene disruption protocol for this pathogen. Our recently established gene deletion protocol [39] makes genetic manipulation of *B. dothidea* possible. In this study, we investigate the mechanism of warts formation on apple shoots and explore the role of a cutinase gene in the interaction between *B. dothidea* and the apple (*Malus domestica*) plant.

## 2. Results

### 2.1. Invasion of B. dothidea Stimulated the Plant Defense Response and Wart Formation

**Figure 1:**
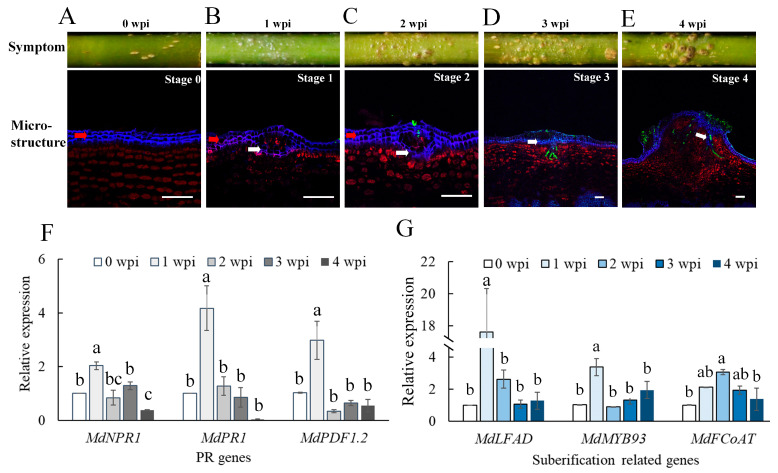
Wart developed on inoculated apple shoots and the infection process of *B. dothidea*. (**A**) Stage 0: the phellem cells (in blue color) and phelloderm cells (in red color) orderly arranged in shoot periderm. (**B**) Stage 1: a new phellem layer formed below the first phellem layer, and the phelloderm cells aggregated disorderly underneath the newly formed (second) phellem layer, indicating the formation of an infection site. (**C**) Stage 2: invasion hypha (green) penetrated the elevated first phellem layer, but not yet the second phellem layer. (**D**) Stage 3: invasion hypha penetrated the second phellem layer and started to extend in phelloderm. (**E**) Stage 4: invasion hypha extended extensively in the phelloderm. The cells around the infection sites significantly proliferated, and suberification was generated in cells surrounding the invasion hypha. The white arrow indicated the second phellem layer. Bar = 50 μm. (**F**) qRT-PCR analysis of pathogenesis-related (PR) genes of *M. domestica* (*MdNPR1, MdPR1, MdPDF1.2*). (**G**) qRT-PCR analysis of suberification related genes (*MdLFAD, MdMYB93, MdFCoAT*) that are involved in the suberification process of *M. domestica*. Samples were prepared for transcript-level analysis from 1 to 4 weeks post-inoculation (wpi), and sample 0 wpi was used as control. Bars represent the standard error of values from two independent experiments. Statistical significance was analyzed by one-way analysis of variance (ANOVA) and Duncan’s test, different letters indicate the significant difference (*p* < 0.05).

To investigate the wart formation, we inoculated the unwounded apple shoots with mycelial blocks of *B. dothidea,* and warts formed on the inoculated shoots around 30 dpi (Figure 1 and Figure S1B). The ultrastructure of wart transactions showed that the wart tissues consist of the phellem layers, the invading mycelia, and the proliferous phelloderm cells underneath the newly formed phellem layer. To study the infection process of *B. dothidea* and the plant responses, we observed the transections of the inoculated sites at various time points post-inoculation and designated five infection stages based on the characteristics observed in the infected plant tissue. At Stage 0, the periderm of intact shoots consisted of an orderly arranged epidermis, phellem, phellogen (cork cambium), phelloderm (parenchyma-like cells) (Figure 1A and Figure S1C). At stage 1, a new phellem layer formed below the first phellem layer, and phelloderm cells aggregated disorderly beneath the newly formed phellem layer, while the invading hypha had not penetrated the first phellem layer. This stage was commonly observed in sections of the inoculation site one-week post-inoculation (wpi) (Figure 1B). When infection reached this stage, we designated that an infection site was successfully generated. At stage 2, the invasion hyphae penetrated the elevated first phellem layer but not yet the newly formed second phellem layer. This stage was commonly observed in inoculation sites at 2 wpi (Figure 1C and Figure S1D). At stage 3, the invading hypha penetrated the second phellem layer and started to expand in the phelloderm. This stage was commonly observed in inoculation sites at 3 wpi (Figure 1D). At stage 4, the invading hypha extended extensively into the phelloderm. The cells beneath the infection sites proliferated, and suberification was generated in cells surrounding the invading hypha (Figure 1E).

To analyze the plant response to pathogen penetration, we tested the expression levels of the host pathogenesis-related genes (*MdNPR1, MdPR1,* and *MdPDF1.2*) and the suberification-related genes (*MdLFAD, MdMYB93,* and *MdFCoAT*) (Table S2) in the cortex tissues at various time points of the infection process. The qRT-PCR analysis showed that all these genes were significantly upregulated at 1 wpi (Figure 1F,G), especially the suberification-related gene *MdLFAD,* which was upregulated about 20 times. At this infection stage, the pathogen hyphae had not yet penetrated the cortex, but a new phellem layer formed, indicating that the *B. dothidea* invasion stimulated the plant defense responses to generate a phellem layer. As cuticle is a physical barrier preventing pathogen invasion of the plant [18], we speculated that fungal cutinase, which can digest cutin, the main constituent of the plant cuticle, may play an important role in the pathogen penetration.

### 2.2. Cutinase Genes in B. dothidea Were Upregulated during Infection of Apple Shoots

**Figure 2:**
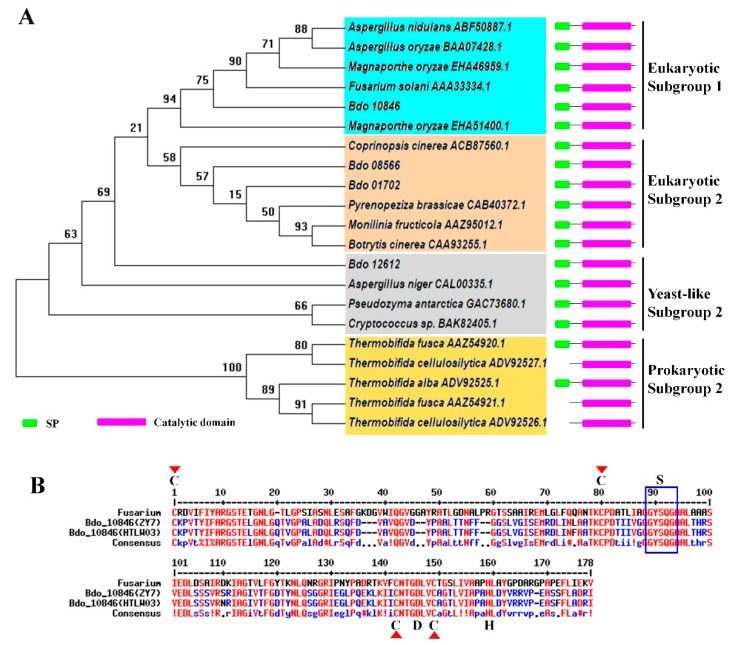
Bioinformatics assay of four *Botryosphaeria dothidea* cutinases that were up-regulated during the infecting process. (**A**) Phylogenetic analysis of four cutinases of *B. dothidea*. A maximum-likelihood tree was constructed using MEGA 7.0 with amino acid sequences of cutinases domain. The statistical strengths were assessed by bootstrap with 1000 replicates. Bootstrap values are shown near the tree nodes. (**B**) The amino acid sequence alignment of *Bdo_10846* domains from two *B. dothidea* strains and the reference sequence of cutinase from *Fusarium solani.* The rectangle indicated the conserved GYSQG catalytic site while S, D, and H were components of the S–H–D catalytic triad motif. Red triangles indicated the conserved amino acid that are important for the spatial conformation of cutinase.

To find cutinase genes that are involved in the *B. dothidea* invasion, we re-investigated 4 of 13 candidate cutinase genes that were detected in the transcriptome of *B. dothidea* infected apple shoots. These cutinase candidates belong to the α/β hydrolase superfamily, contain a signal peptide, have less than 400 amino acids, and are cysteine-rich proteins except Bdo_04657 (Table S1). Phylogenetic analysis showed that the *Bdo_10846*-encodes a protein that belongs to the cutinase of Eukaryotic Subgroup 1, which encompasses many cutinases associated with pathogen virulence or plant defense responses. *Bdo_01702* and *Bdo_08566* encode the cutinases belonging to Eukaryotic subgroup 2, while *Bdo_12612* encodes a cutinase belonging to the yeast-like subgroup (Figure 2A).

Protein structure analysis suggested that the *Bdo_10846* of two *B. dothidea* strains (HTLW03 and ZY7) had a conserved GYSQG catalytic site and one S–H–D catalytic triad motif (Figure 2B). Quantitative RT-PCR analysis revealed that the expression of *Bdo_10846* was upregulated from 1 wpi to 3 wpi with the highest expression levels at 1 wpi (Figure S2), which was coincident with the upregulation of the suberification-related genes, indicating that *Bdo_10846* potentially played an important role in the interaction between *B. dothidea* and its host. Thus, we further investigated the biological function of this gene.

### 2.3. Enzyme Activity, Signal Peptide Examination and Localization of Bdo_10846

**Figure 3:**
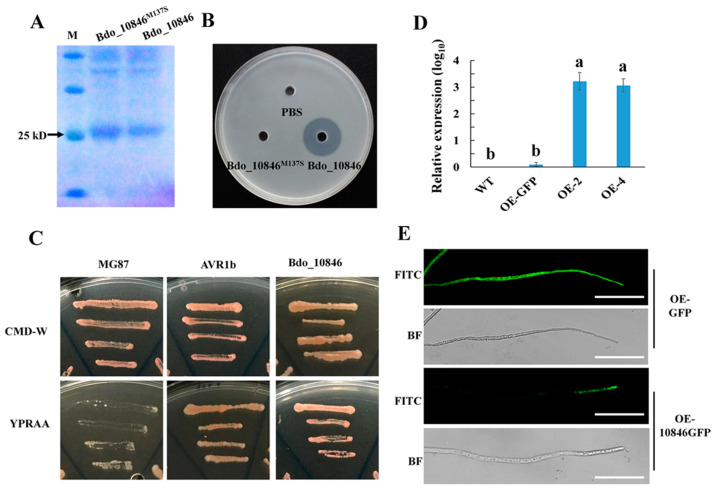
Cutinase activity, signal peptide (SP) activity, and localization of Bdo_10846. (**A**) Purification of the prokaryotic expressed Bdo_10846 and Bdo_10846^M137S^ (a site-directed mutant protein, in which the serine in catalytic activity center was replaced by alanine), using affinity chromatography. (**B**) Validation of cutinase Bdo_10846 activity with medium containing tributyrin, an insoluble ester. When tributyrin was degraded, a transparent zone was generated. (**C**) Validation of the secretion function of the Bdo_10846 signal peptide using the yeast signal trap assay. The functional signal peptide of AVR1b was used as a positive control, while the unsecreted MG87 was used as a negative control. (**D**) The expression level of *Bdo_10846* in two overexpression transformants—OE-2 and OE-4. Bars represent the standard error of values from two independent experiments. Statistical significance was analyzed by one-way analysis of variance (ANOVA) and Duncan’s test, different letters indicate the significant difference (*p* < 0.05). (**E**) Localization of Bdo_10846 fusion protein Bdo_10846GFP in *B. dothidea* mycelia was observed under a fluorescence confocal microscope, Bar = 50 μm.

To investigate if Bdo_10846 has cutinase activity, plasmids expressing His-tagged wild type Bdo_10846 and a mutant protein bearing M137S at the catalytic site were constructed, resulting in proteins with a molecular size of 25 kD (Figure 3A). The *Escherichia coli* strains expressing individual proteins were cultured in a medium containing triglyceride, a common substrate of cutinase, on which a transparent zone will appear when the triglyceride is hydrolyzed. The assay showed that the fusion protein 6×His-Bdo_10846 had a strong hydrolytic activity to triglyceride, whereas, the site-directed mutant protein 6×His-Bdo_10846 ^M137S^ lost cutinase activity (Figure 3B). This result demonstrated that Bdo_10846 had high cutinase activity and the Ser-137 catalytic site was essential for its catalytic function.

To investigate if Bdo_10846 can be secreted by *B. dothidea*, the yeast secretion trap assay (YST) was carried out. The result showed that yeast containing the reporter gene *suc2*, fused with the signal peptide (SP) of Bdo_10846, could grow well on both CMD-W and YPRAA media, whereas the *suc2* without the SP sequence only grew well on CMD-W but not on YPRAA media. This indicated that the SP of Bdo_10846 had secretion activity and Bdo_10846 was a secreted protein (Figure 3C).

To observe the localization of Bdo_10846 in the pathogen, we fused the synthetic green fluorescent protein (sGFP) to the Bdo_10846 C-terminus and generated two over-expression transformants (OE-2 and OE-4). The expression levels of *Bdo_10846* in the transformants were significantly higher than that in the control strain (OE_GFP transformant), and the green fluorescence accumulated at the tip of hyphae in the *Bdo_10846* over-expressing transformants, but not in the control strains (Figure 3D,E), suggesting that Bdo_10846 mainly located in the tip of hyphae.

### 2.4. Deleting Bdo_10846 Reduced the B. dothidea Cutinase Activity

To explore the biological function of Bdo_10846 in *B. dothidea*, we generated *Bdo_10846* knockout transformants by replacing the ORF fragment with *hph* through homologous recombination. We obtained two *Bdo_10846* knockout transformants (*ΔBdo_10846-3 and ΔBdo_10846-6*), which were verified by three-step PCR and southern-blot analyses (Figure S3). Similarly, we generated two *Bdo_10846* complementary transformants (C-1 and C-3). The *Bdo_10846* knockout transformants had similar growth rates, conidia morphology, conidia production ability, and conidia germination rates as the wild type (WT) strain and the complementary transformants on potato dextrose agar (PDA) (Figure S4). However, the *Bdo_10846* knockout transformants had a reduced growth rate and lower wet weight in cutin broth (cutin as the sole carbon source) compared with the WT strain and the complementary transformants (Figure S5A,B). Moreover, cutinase activity in the cultural broth was analyzed using p-nitrobenzoate (PNB). The result showed that cutinase activity in the cultural broth of two *Bdo_10846*-knockout transformants significantly decreased (Figure S5C,D). These results showed that deleting *Bdo_10846* decreased the cutinase activity of *B. dothidea*.

### 2.5. Bdo_10846 Is Required for the Full Virulence of B. dothidea

**Figure 4:**
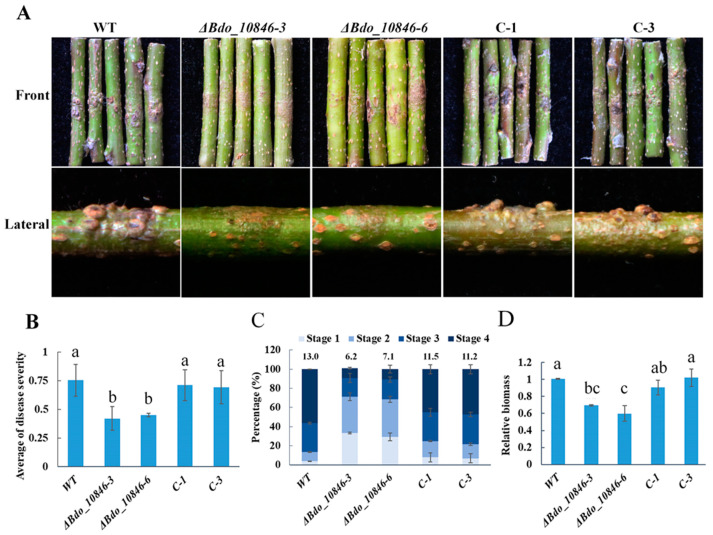
Pathogenicity assay of *Bdo_10846* transformants on apple shoots. (**A**) Warts formation on shoots (cv. Fuji) inoculated with wild type (WT), knockout transformants *(ΔBdo_10846-*3, *ΔBdo_10846-*6*),* and complementary transformants. Images were taken at 5 wpi. (**B**) Statistical results of disease severity. (**C)** Statistical results of infection sites at various stages. The inoculation sites were transected using freezing-microtome, stained with FITC-wheat germ agglutinin (F-WGA), and observed under a fluorescence microscope. (**D**) The biomass of invasion hyphae in tumors was determined using qRT-PCR. The data were average (and standard error) of values from two independent experiments. Statistical significance was analyzed with ANOVA and Duncan’s test, different letters indicate the significant difference (*p* < 0.05).

To determine if Bdo_10846 is required for the pathogenicity of *B. dothidea*, WT strain and its transformants were inoculated on apple shoots. The disease severity of *Bdo_10846*-knockout transformants (*ΔBdo_10846-3* and *ΔBdo_10846-6*) was significantly decreased compared with the WT and the complementary strains. (Figure 4A,B). We further explored the infection of the *Bdo_10846* knockout transformants via observing the cross-sections of the inoculation sites. The average amount of infection sites on cross-sections was 6.2 (*ΔBdo_10846-3*) and 7.1 (*ΔBdo_10846-6*) for *Bdo_10846* knockout transformants, respectively, whereas it was 11.5 (C-1), 11.2 (C-2), and 13 (WT) for the complementary and WT strains, respectively. Additionally, around 30.04% of infection sites caused by *ΔBdo_10846-3* and *ΔBdo_10846-6* were at the infection stage 3 and 4, while, 80% to 86% of infection sites caused by complementary and WT strains were at infection stage 3 and 4 (Figure 4C). Furthermore, the qRT-PCR analysis revealed a significant decrease of *B. dothidea* biomass in warts induced by *Bdo_10846* knockout transformants compared with those in warts caused by complementary and WT strains (Figure 4D). These results suggested that *Bdo_10846* is required for the full virulence of *B. dothidea*.

### 2.6. Bdo_10846 Can Trigger Plant Defense Responses

**Figure 5:**
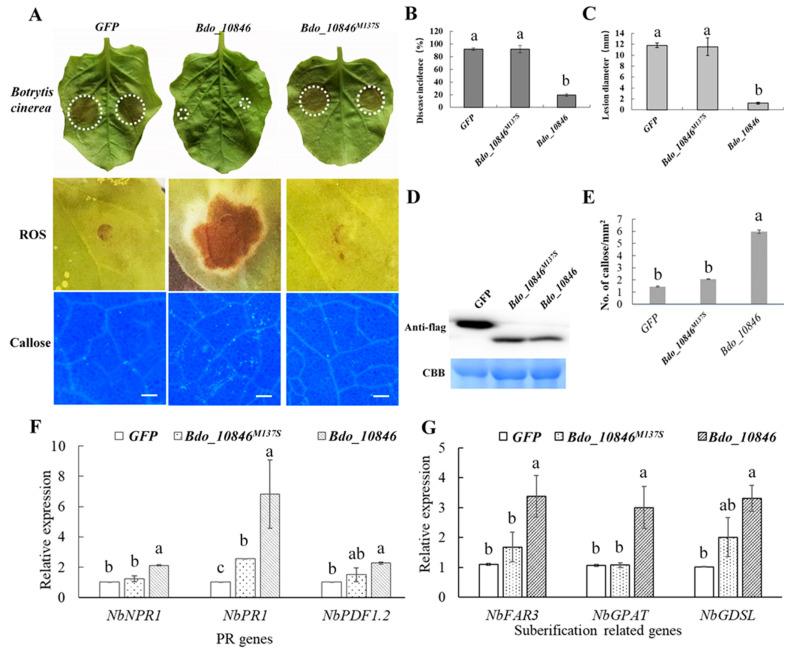
Transient expression assay of *Bdo_10846* in *Nicotiana benthamiana.* (**A**) The Reactive oxygen species (ROS) burst and callose accumulation 48 h after transiently expressing genes (*GFP, Bdo_10846^M137S^, Bdo_10846*). Image of *N. benthamiana* leaves surveyed 72 h after inoculating *Botrytis cinerea*, Bar = 200 μm. (**B**,**C**) Statistical results of disease incidence and lesion diameters on *N. benthamiana* 72 h after inoculating *B. cinerea* on the leaves expressing genes *GFP, Bdo_10846^M137S^, Bdo_10846*. (**D**) Western blot analysis of proteins (GFP, Bdo_10846^M137S^, Bdo_10846). (**E**) Statistical result of callose formation in *N. benthamiana* leaves. (**F**,**G**) Expression levels of pathogenesis-related (PR) and suberification related genes in *N. benthamiana* 48 h after transient expression of *Bdo_10846*. Bars represent the standard error of values from two independent experiments. Statistical significance was analyzed with ANOVA and Duncan’s test, different letters indicate the significant difference (*p* < 0.05).

Previous studies have revealed that cutinases of some plant pathogens can induce plant defense responses and are involved in innate immune recognition [34,40]. To determine whether Bdo_10846 is involved in the interaction between *B. dothidea* and plants, a transient expression assay was performed in *N. benthamiana*. Transient expression of Bdo_10846, but not Bdo_10846^M137S^ or GFP, resulted in ROS burst and callose deposition in *N. benthamiana* 48 h post infection, and a decrease in disease incidence and lesion diameter caused by *B. cinerea* (Figure 5A–C). Additionally, the qRT-PCR assay revealed the upregulated expression of pathogenesis-related genes *NbNPR1*, *NbPR1*, and *NbPDF1.2*, in addition to the suberification-related genes (Table S2) in *N. benthamiana* transiently expressing Bdo_10846 but not Bdo_10846^M137S^ (Figure 5D–G). These results suggested that Bdo_10846 could induce the plant defense response, and the enzymatic activity was essential for the induction.

### 2.7. NbBAK1 and NbSOBIR1 Are Required for Bdo_10846 Induced Defense Responses

**Figure 6:**
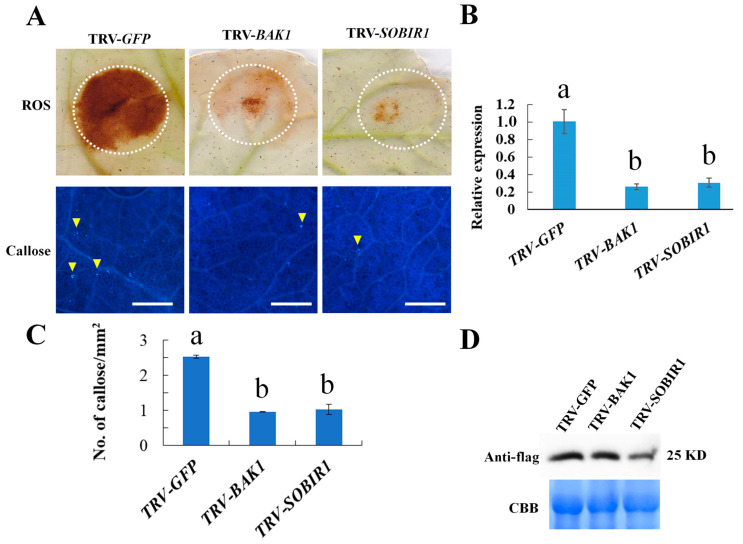
Defense response of *N. benthamiana* required NbBAK1 and NbSOBIR1. (**A**) Callose and H_2_O_2_ accumulation in control (TRV-GFP) and *NbBAK1* (TRV-*BAK1*) and *NbSOBIR1* (TRV-*SOBIR1*) silenced *N. benthamiana* leaves. Virus-induced gene silencing (VIGS) was used for silencing *NbBAK1* and *NbSOBIR1*. (**B**) Silencing efficiency of immunity co-receptor genes *NbBAK1* and *NbSOBIR1* detected using qRT-PCR. Data were the average and standard error of two independent experiments. (**C**) Amount of callose accumulation. Data were the average (and standard error) of two independent experiments. (**D**) Western blot analysis of cutinase Bdo_10846 in *N. benthamiana* 48 h after transient expression. Statistical significance was analyzed with ANOVA and Duncan’s test, different letters indicate the significant difference (*p* < 0.05).

To investigate if co-receptors NbBAK1 and NbSOBIR1 of DAMPs recognition were involved in defense responses induced by Bdo_10846, we transiently expressed Bdo_10846 in the *NbBAK1* and *NbSOBIR1* silenced *N. benthamiana* leaves. Cutinase Bdo_10846 induced ROS burst and excessive callose deposition in control plants, but not in *NbBAK1* and *NbSOBIR1* silenced *N. benthamiana* plants (Figure 6). This result suggested that NbBAK1 and NbSOBIR1 are required for Bdo_10846 to induce defense responses in *N. benthamiana*.

## 3. Discussion

*B. dothidea* can cause various symptoms on apples, including warts and cankers on twigs, branches, and stems. Recently, 320 candidate effectors in the *B. dothidea* genome were predicted [41]. However, the mechanisms of wart formation and the pathogenesis of *B. dothidea* are not clear. Results of this study showed that the wart on shoot induced by *B. dothidea* consists of proliferous phelloderm cells, the newly formed phellem layer, and the suberized phelloderm cells surrounding the invading mycelia. The qRT-PCR analysis showed that the *B. dothidea* stimulated the upregulation of the apple pathogenesis-related genes and the suberification-related genes. These results showed that the proliferation of phelloderm cells and the formation of the phellem layer in response to the *B. dothidea* invasion led to the formation of wart symptom on apple shoots and wart formation is a resistant response of plants to the invasion of *B. dothidea*. Additionally, the active growth of the plant is essential for wart formation. This resistant response may be related to the cork cambium development. Generally, woody plants have phellogen (cork cambium) in their stems and roots, and the meristematic cell layer of cork cambium forms phellem (cork) centrifugally and phelloderm (parenchyma-like cells) centripetally [42]. The phellem layer with suberin deposition in the cell wall protects the stem from water loss and mechanical damage including pathogen invasion. Taken together, this can explain the observation that the wart formation is associated with the growth stage and the condition of the plant [6]. A previous study showed that warts commonly formed around 30 dpi on intact shoots that were inoculated without wounding during the period from May to July in northern China, but not on shoots of a plant with drought stress. Instead, canker symptom was observed [6]. Additionally, warts commonly develop in the following year but not the same year on shoots that were inoculated in August or later [6].

In this study, we also identified a cutinase gene of *B. dothidea* that encodes a secreted cutinase of 25 KD, which showed significant upregulation coincident with the upregulation of the suberification-related genes. The *Bdo_10846* knockout transformants had reduced cutinase activity and weakened virulence compared with the WT and complementary strains, suggesting that *Bdo_10846* is required for the full virulence of *B. dothidea*. Additionally, the *Bdo_10846* knockout transformants also showed a slower growth rate in cutin broth than WT and complementary transformants. Previously, studies have demonstrated that cutinases are associated with carbon acquisition [28,43]. Cutinases can efficiently hydrolyze cutin and suberin [18,44,45,46]. In this study, the *Bdo_10846* knockout transformants showed a slower growth rate in cutin broth than WT and complementary transformants. Therefore, the decrease in the virulence of *Bdo_10846* knockout transformants may attribute partially to the reduced hyphae expansion due to the decrease in carbon acquisition from plant tissue containing cutin and suberin.

**Figure 7:**
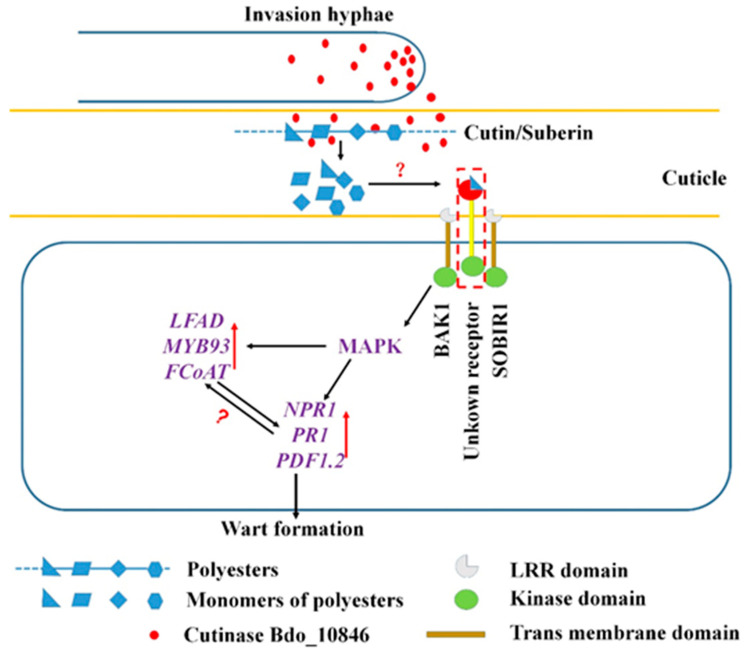
A model of warts formation on apple shoots induced by *B. dothidea* invasion. Upon invading apple shoot, *B. dothidea* released cutinase Bdo_10846 to digest cutin and suberin in epidermal and phellem cells. Unknown monomers released from hydrolyzing the cutin and suberin were recognized by a receptor and triggered the defense response through the (mitogen-activated protein kinase (MAPK) pathway, inducing the subsequent upregulation of pathogenesis-related (PR) gene and suberification-related (SR) genes. As a result, the phellogen formed phelloderm outward and phelloderm inward around the infection site to resist the invading of the pathogen, and suberification was generated in the phelloderm cells surrounding the invading hypha.

The oligogalacturonans (OGs), a type of DAMPs can be released when homogalacturonan (the major component of pectin) is cleaved by polygalacturonic acid endonuclease [33,47]. Receptor-like kinases (RLK) and receptor-like proteins (RLP) are key PRRs for the recognition of PAMPs and DAMPs. Previous studies have identified the OGs receptor (WAK1), PEPs receptor (PEPR 1/2), and PIPs receptor (RLK7) [32]. In addition, the brassinosteroid insensitive 1-associated receptor kinase 1 (BAK1) and the suppressor of the interaction between BAK1 and RLK (SOBIR1) serve as co-receptors of the immune receptor complex involved in DAMPs recognition [48,49]. In this study, we demonstrated that the enzyme activity is essential for the cutinase Bdo_10846 to induce the resistance of *N. benthamiana* to *B. cinerea*. Furthermore, our results also showed that *NbBAK1* and *NbSOBIR1* were required for Bdo_10846-induced defense response in *N. benthamiana*. In *Arabidopsis thaliana*, cutinase-expressing plants (CUTE plants), such as some cuticle-deficient mutants *bdg*, *eca2*, and *ohy1*, are resistant to *B. cinerea* [34,50,51]. Our finding is similar to what has been found for cutinase VDCUT11 of *V. dahlia*, which was suggested to act as a DAMP [35], but is different from the cutinase SsCUT of *Sclerotinia sclerotiorum*, a PAMP whose enzyme activity is dispensable for triggering plant defense responses [47]. Previous studies showed that free cutin monomers released from cutinase degradation by plant pathogen could serve as DAMPs [52,53]. Both cutin and suberin are polyester and can be hydrolyzed by cutinase [18,35]. Therefore, we suspected that some molecules, released from the hydrolysis of cutin, suberin, or other esters in the cuticle and phellem layers of apple shoots, might serve as DAMPs in triggering the plant defense response (Figure 7). Therefore, if these DAMPs are absent, in the case of pathogen invading through wounds without digesting phellem layers, the Bdo_10846-induced wart formation will be absent as well. This explains the previous observation that warts commonly formed on intact shoots inoculated without wounding, but canker symptom forms on shoots inoculated with wounding [6]. However, further investigations are needed to reveal the specific molecules and receptors involved.

*B. dothidea* can infect hundreds of plant species serving as an endophyte or pathogen [1]. Previous studies have shown that the amount of CAZymes in plant pathogenic fungi was related to their host range [54]. The fungi with dual lifestyles as endophyte and pathogen have numerous highly diverse CAZymes as they have to adapt to utilize the limited intercellular nutrients from plant tissue [55]. In the *B. dothidea* genome, 13 cutinase genes were identified, but only a few were upregulated in the process of infecting apple shoots [38]. Variation in biological function has been found in cutinases of *V. dahliae* [35]. Therefore, different cutinases may play different biological roles in infecting different plant species in addition to the functional redundancy. Further investigations on the infection of *B. dothidea* on various hosts may unveil their biological functions. Taken together, the results of this study demonstrated that the cutinase Bdo_10846 was required for the full virulence of *B. dothidea* and played important roles in triggering the plant defense responses and the formation of wart symptom. Our findings reveal the mechanism of wart formation and provide new insights into the pathogenicity mechanism of *B. dothidea*. *B. dothidea* is the type species of *Botryosphaeria* (Botryosphaeriaceae, Botryosphaeriales), which contains many widespread and important woody plant pathogens. Results of this study will benefit the understanding of the pathogenesis of *B. dothidea* on other woody plants and the pathogenesis of many important pathogens in Botryosphaeriaceae.

## 4. Materials and Methods

### 4.1. Isolates Used and Culture Conditions

The virulent *B. dothidea* strain, HTLW03, was isolated from crabapple, and the *B. cinerea* strain SCCD2 was isolated from grape and stored in the Mycology lab, China Agricultural University, Beijing, China. The *B. dothidea* isolate and its transformants were grown and maintained on potato dextrose agar (PDA) plate at 26 °C, while the *B. cinerea* strain SCCD2 was maintained on PDA plate at 20 °C in darkness. To test the mycelial linear growth rate of *B. dothidea* strains, culture plugs (5 mm in diameter) were cut from the edges of the actively growing cultures, placed on PDA plates, and incubated at 26 °C for 60 h.

To induce the conidia formation of *B. dothidea*, the aerial mycelium of three-day-old colonies on PDA plates were scrapped with a scalpel and the plates were then incubated at 26 °C under the blacklight for 20 days [56]. Mature pycnidia of the isolates were collected and crushed in a micro-centrifuge tube with 0.5 mL sterile distilled water. Three 90-mm plates were used for each strain and the experiment was repeated once. To induce the conidia formation of *B. cinerea,* strain SCCD2 was grown first on PDA plate at 20 °C in darkness for 72 h. Then, the culture was incubated under the blacklight for 20 days for sporulation. Distilled water was added to plates to make conidia suspension. The concentration of conidia suspensions was measured with a hemocytometer.

### 4.2. Analysis of Cutinase Protein and Gene Sequences

Sequences of cutinase were retrieved from the NCBI database. Structural domains of candidate cutinases were predicted using the NCBI Conserved Domain Search Tool (https://www.ncbi.nlm.nih.gov/Structure/cdd/wrpsb.cgi) (Bethesda, MA, USA). The signal peptide was predicted using the signalIP-5.0 online tool (http://www.cbs.dtu.dk/services/SignalP/) (DTU, Copenhagen, Denmark). The subcellular localization of proteins was predicted using WoLF PSORT (https://www.genscript.com/wolf-psort.html) (Kenta Nakai, University of Tokyo, Tokyo, Japan) and Euk-mPLoc 2.0 (http://www.csbio.sjtu.edu.cn/bioinf/euk-multi-2/) (Shanghai Jiao Tong University, Shanghai, China)**.** The promoter sequence was predicted using Berkeley Drosophila Genome Project tool (http://www.fruitfly.org/seq_tools/promoter.html) (Drosophila Genome Center, Bethesda, MD, USA). Finally, the transmembrane domains were predicted using TMHMM Server, v. 2.0 (http://www.cbs.dtu.dk/services/TMHMM/) (DTU, Copenhagen, Denmark) [36]. A phylogenetic tree was constructed with the maximum likelihood evolution algorithm in Molecular Evolutionary Genetics analysis (MEGA) 7.0 (Sudhir Kumar, Arizona State University, Knicks, AZ, USA) [57]. A Poisson correction was used for multiple substitution models and pairwise deletion was used for gap split data treatment. The statistical strengths were assessed by bootstraps with 1000 replicates.

### 4.3. RNA Extraction and qRT-PCR

To detect the expression pattern of genes in infected tissues, the bark tissues (0.5 × 0.5 cm) from five inoculation sites were collected at 0, 1, 2, 3, and 4 weeks after inoculation. To detect the expression levels of transient expressed *Bdo_10846* in *N. benthamiana*, six-leaf discs (1 cm diameter) were collected at 48 h after infiltration. Total RNA of *M. domestica* bark tissues and *N. benthamiana* leaves was extracted using the Nucleospin RNA Plant kit (MACHEREY-NAGEL., Düren, Germany). The concentration and quality of RNA was analyzed using a Nanodrop 2000 Spectrophotometer (Thermo Fisher Scientific, Waltham, MA, USA) before the RNA samples were reversely transcribed with an oligo (dT)_18_ primer using Reverse Transcriptase M-MLV (TaKaRa Bio Inc. Shiga, Japan) following the manufacturer’s instruction.

The *B. dothidea Actin*, *M. domestica GADPH*, and *N. benthamiana* elongation factor (EF1α) gene were used as internal control, respectively [15]. The PCR was performed in qPCR Tower2.0 (Analytik, Jena, Germany) using TB Green Premix DimerEraser^TM^ qPCR mix (TaKaRa, Dalian, China) with primers listed (Table S3). The results of qRT-PCR (relative expression level and relative biomass) were analyzed using the 2^−ΔΔct^ method [58]. Means were calculated using data from three replicates. Experiments were repeated with a different set of biological samples.

### 4.4. Plasmid Construction

To construct *Bdo_10846* deletion vector, the 1-kbp of 5′and 3′ flanking fragments were amplified from *B. dothidea* HTLW03 genomic DNA, respectively. The two fragments were ligated to the 5′and 3′ends of 1.8 kb hygromycin-resistant gene (*hph*) by double-joint PCR [59] and introduced into the pMD19-t vector using T/A ligation kit (TaKaRa, Dalian, China). Similarly, the *NPT*II cassette was constructed and ligated into the pMD19-t vector site.

The complementary fragment including the open reading frame of *Bdo_10846* with approximately 1500 bp promoter and 500 bp terminator was amplified from the *B. dothidea* genomic DNA and inserted into the pMD19-T plasmid at the *Hind*III site. Similarly, the *Histone 3* promoter (*H3*) of *B. dothidea* was amplified with primer pair H3-F/H3-R.

The plasmid pSilent1 was used to construct the *Bdo_10846* overexpression vector. The *Trp*C promoter in pSilent1 was cut off by *Sal*I (TaKaRa, Dalian, China) and replaced with *H3*. The primary cutinase intron was cut off by *Xhol*I and *Bgl*II (TaKaRa, Dalian, China) and replaced with the *sGFP* fragment that was amplified with primer pair 10846OE-F/10846OE-R from cDNA of *B. dothidea* HTLW03. Then, the CDS fragment of *Bdo_10846* without stop codon was ligated into the N-terminus of the sGFP at *the Hind*III site (Figure 7 and Figure S2). Ligations of plasmids except for the T/A clone were performed using a one-step-clone kit (Vazyme, Nanjing, China); primers used in this study were listed in Table S4. The generation of transformants was performed as described by Dong and Guo [39].

### 4.5. Yeast Secretion Trap Assay

The secretion activity of Bdo_10846 was analyzed using yeast secretion trap assay following the description of Jacobs et al. [60]. Briefly, the predicted signal peptide (SP) of Bdo_10846 was fused to N-terminal of secretion-defective invertase gene (suc2) in the vector pSUC2 and transformed into the yeast strain YTK12 using T2001Frozen-EZ Yeast Transformation II Kit (Zymo Research, Irvine, CA, USA). The transformants were selected on CMD-W (lacking tryptophan) medium. Then, the positive transformants were incubated on YPRAA (2% raffinose) [61]. The SPs of Avr1b and MG87 were used as the positive and negative control, respectively.

### 4.6. Pathogenicity Test

The pathogenicity test was conducted on new shoots of 9- to 10-year-old apple trees (*M. domestica* “Fuji”) from May to July in 2018 and 2019 in an orchard in Beijing using the method described by Tang et al. [6]. For each tested strain, five inoculations were made on a shoot, six shoots on a tree were used as a replicate, and three replicates were used for each strain in a completely random design. All experiments were repeated as indicated. Disease severity was recorded on a scale from 0 to 4 based on the number of warts on the inoculation site, following the description of [62] with some modification. Briefly, 0: no tumor; 1: 1–5 visible tumors (the height is higher than 0.5 mm); 2: 6–10 visible tumors; 3: 11–15 visible tumors; 4: more than 15 visible tumors. The average of diseased severity was calculated to represent the disease severity caused by each isolate.

### 4.7. Histochemical Analysis

To analyze the infecting process of WT in plant tissues, inoculated shoot samples were taken at 0, 1, 2, 3, and 4 weeks post inoculation. A 0.5-cm-long segment containing the original inoculation spot was excised from each inoculation site and preserved in 4% paraformaldehyde (Solarbio, Beijing, China) for 12 h. The samples were transferred to a flask with a 5-mL solution containing 10% glycerol (v/v) and 20% sucrose (m/v) and were preserved under a vacuumed condition for 6 h. Then, the segments were placed in optimal cutting temperature (OCT) compound (SAKURA, Tokyo, Japan), at −30 °C for 10 min and sectioned transversely using Leica microtome CM1850 (Leica, Weztlar, Germany) [63].

The sliced tissues were stained with FITC-wheat germ agglutinin (F-WGA, Sigma, Saint Louis, MO, USA) following the protocol of Inch, et al. [64] with some modifications. Briefly, the cross-sections were washed three times with 0.05% Tween 20, soaked into 10% KOH solution for 10 min, washed with 0.1 M phosphate buffer saline (PBS) solution (pH = 7.2), and soaked in bovine serum albumin (BSA, Sigma, Saint Louis, MO, USA) solution (20% BSA, 0.1 M PBS pH = 7.2) for 10 minutes before being stained with F-WGA (Sigma, Saint Louis, MO, USA) solution (0.01% F-WGA, 1% BSA, 0.1M PBS, pH = 7.2) for one hour. The invasion hypha was observed with a green light (exciter 488 nm and 495–545 nm absorption) [63], and the cortex tissues were observed under blue light (with 405 nm exciter and 410–480 nm absorption) [17,65]. For each strain, three replicates were included for each time point. Each replicate contained four samples that were taken from two inoculated sites of two different inoculated shoots, and two sections from each sample were surveyed. The experiments were conducted in 2018 and repeated in 2019.

### 4.8. Prokaryotic Expression of Bdo_10846

The wild-type gene *Bdo_10846* and mutated gene *Bdo_10846^M137S^* (Serine-137 was replaced with alanine) with a 6×His tag on the N-terminus were ligated into the pHAT vector and transformed into *Escherichia coli BL21*(DE3) pLysS (ZOMANBIO, Beijing, China). The expression of the recombinant proteins was induced by adding 0.5 mM isopropyl-β-D-thiogalactoside for 12 h at 18 °C. The cells were then lysed by sonication, and the lysate was centrifuged for 20 min at 20,000 *g*. Then, the proteins in the supernatant were purified by affinity chromatography [66].

### 4.9. Cutinase Activity Assay

The cutinase activity of Bdo_10846 and Bdo_10846^M137S^ was tested using the method described by Kwon et al. [21] with some modification. Briefly, three holes (5 mm in diameter) were punched on the tributyrin plate (0.5% tributyrin, 0.05M PBS, pH = 7.0, 1% agar) with a hole puncher, and 100 ng cutinase in PBS (pH = 7.0) was added in each hole. The transparent zones around each hole were observed after 12-h incubation at 26 ℃ in darkness.

The cutinase activity of WT and *Bdo_10846* transformants were evaluated by testing their carbon acquisition from tomato cutin. A 0.5-cm culture plug was cultured in 5 mL cutin broth (NaNO_3_ 0.3%, K_2_HPO_4_ 0.1%, KCl 0.05%, FeSO_4_·7H_2_O 0.001%, tomato cutin 0.2%) at 26 ℃ in darkness for 10 days, then the liquid culture was centrifuged for 5 min at 5000 *g*. The resulted pellet was weighed and the supernatant was evaluated for the secreted cutinase activity by ρ-nitrophenylbutyrate (PNB) [20]. Three replicates were used for each strain. All assays were performed twice.

### 4.10. Virus-Induced Gene Silencing (VIGS)

VIGS assays were performed as described by Hayward et al. [67]. Briefly, constructed plasmids pTRV1, pTRV2-*BAK1,* and pTRV2-*SOBIR1* were introduced into *Agrobacterium tumefaciens* GV3101. The *Agrobacterium* cells were suspended in MMA solution (10 mM MgCl_2_, 10 mM MES, 100 μM Acetosyringone), and adjusted concentration to 1.0 at OD_600_. Cultures containing the pTRV1 with pTRV2-*BAK1* or pTRV2-*SOBIR1* plasmid were mixed in a 1:1 ratio for infiltration. The mixture was infiltrated into two primary leaves of four-week-old *N. benthamiana* plants. The pTRV2-*GFP* was used as control. Silencing efficiencies of *NbBAK1* or *NbSOBIR1* were validated using qRT-PCR method three weeks after infiltration. Three silenced plants of each gene were used for transient expression of cutinase.

### 4.11. Transient Expression Assay

To determine if Bdo_10846 can induce plant defense response, transient expression of *Bdo_10846* was performed in *Nicotiana benthamiana* following the method described by Legay et al. [15], and the GFP was used as the negative control. Challenging inoculation of *B. cinerea* was performed at 48 h post infiltration. At the same time, ROS burst, callose deposition, and the expression of PR protein were detected. Inoculation of *B. cinerea* [52], diaminobezidin (DAB) staining [68] to detect ROS burst, and the callose deposition [69] were performed. For each gene, 12 leaves from three plants were used for challenging inoculation, and then three leaves were used for callose deposition and ROS burst detection, respectively. The experiments were performed twice.

### 4.12. Statistical Analysis

Analysis of variance (ANOVA) was performed first to test if the effects of treatment/time were statistically significant using Statistical Product and Service Solutions (SPSS) (IBM, Armonk, NY, USA). In case when significant treatment effects were detected, multiple mean comparisons were performed using Duncan’s test. A confidence level of 0.05 was used in all statistical tests.

## Data Availability

The data presented in this study are available in the article.

## Supplementary Materials

The following are available online at https://www.mdpi.com/1422-0067/22/4/1910/s1, Figure S1: Warts on apple shoots induced by *Botryosphaeria dothidea*, Figure S2: Expression pattern of cutinase Bdo_10846, Figure S3: Homologous recombination strategy and verification of the Bdo_10846 knockout transfromants, Figure S4: Mycelial growth and sporulation of Bdo_10846 transformants and WT strains, Figure S5: The mycelial growth and the cutinase activities of Bdo_10846 transformants in medium with cutin as carbon source, Table S1: Information of the predicted 13 cutinases in *B. dothidea*, Table S2: Information of genes involved in the suberin synthesis process. Table S3: Primers used for qRT-PCR, Table S4: Primers used for plasmid construction and transformants verification.
