# Supplementary material for: The Cutinase Bdo_10846 Play an Important Role in the Virulence of Botryosphaeria dothidea and in Inducing the Wart Symptom on Apple Plant
Source: Int J Mol Sci. 2021 Feb 14;22(4):1910. doi: 10.3390/ijms22041910 (PMC7918748; doi:10.3390/ijms22041910)
Supplement: Supplementary file 1 [file ijms-22-01910-s001.pdf]

International Journal of
Molecular Sciences

MDPI

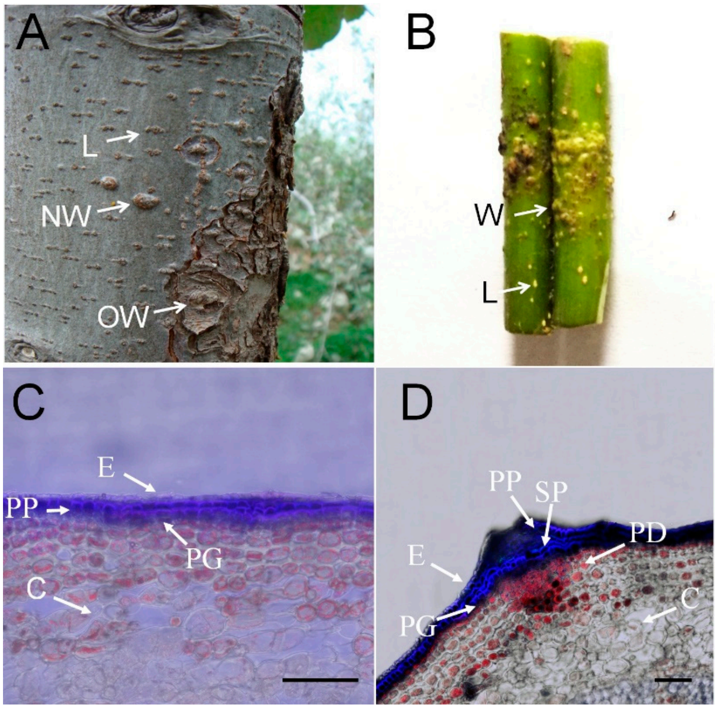

Figure S1. Warts on apple shoots induced by *Botryosphaeria dothidea*. (**A**) Warts on a naturally infected apple branch. NW: newly formed wart; OW: old wart from previous seasons; and L: lenticel. (**B**) Wart on shoots formed 30 days after inoculation. W: wart; L: lenticel. (**C**) Histostructure of periderm of a mock-inoculated apple shoot. E, epidermis (the transparent layer); PP, primary phellem (in blue color), PG, phellogen (cork cambium); PD, phelloderm; and C, cortex. (**D**) Hstostructure of a wart formed on inoculated apple shoot. E, epidermis (the transparent layer); PP, primary phellem (in blue color), SP, newly formed phellem (in blue color); PG, phellogen; PD, phelloderm; and C: cortex. Bar=50 μm.

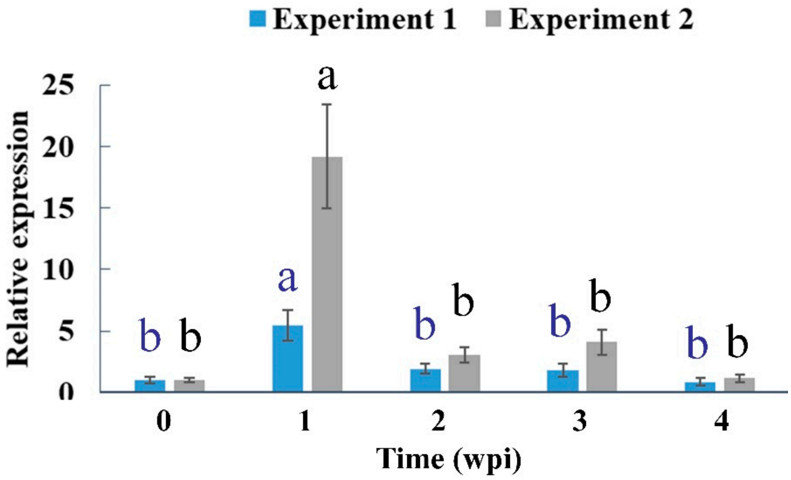

Figure S2. Expression pattern of cutinase gene *Bdo_10846*. Expression level of cutinase *Bdo_10846* at 0, 1, 2, 3, 4 wpi in apple shoots. Data were average and standard deviation of three replicates. Statistical significance was analyzed with one-way analysis of variance (ANOVA) and Duncan´s test, different letters indicate significant difference ($p < 0.05$). Columns and bars represent means and standard errors for three replicates, respectively.

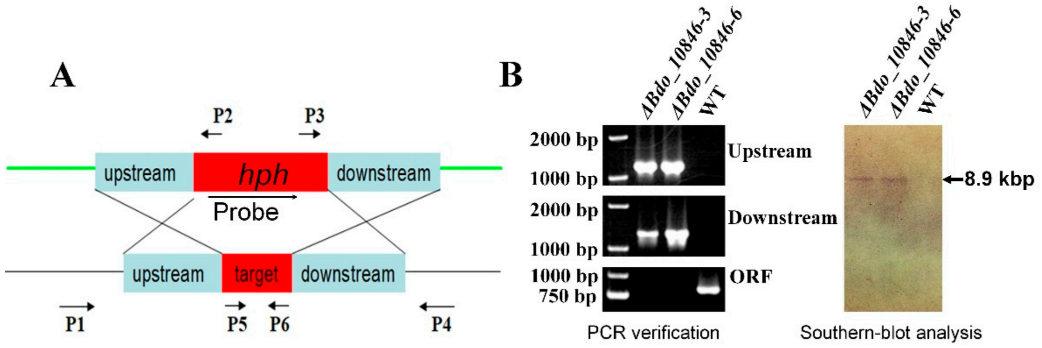

Figure S3. Homologous recombination strategy and verification of the Bdo_10846 knockout transfromants. (**A**) Homologous recombination strategy and primers used for verifying the Bdo_10846 knockout transformants. (**B**) PCR and southern-blot analysis of *Bdo_10846* knockout transformants (*ΔBdo_10846-3* and *ΔBdo_10846-6*). About 1 kb PCR products of upstream (with P1 and P2) and downstream (with P3 and P4) were amplified. The open reading frame fragment (ORF) were absent in the PCR product (with P5 and P6). The 500 bp hph gene fragment was used as probe in southern-blot analysis.

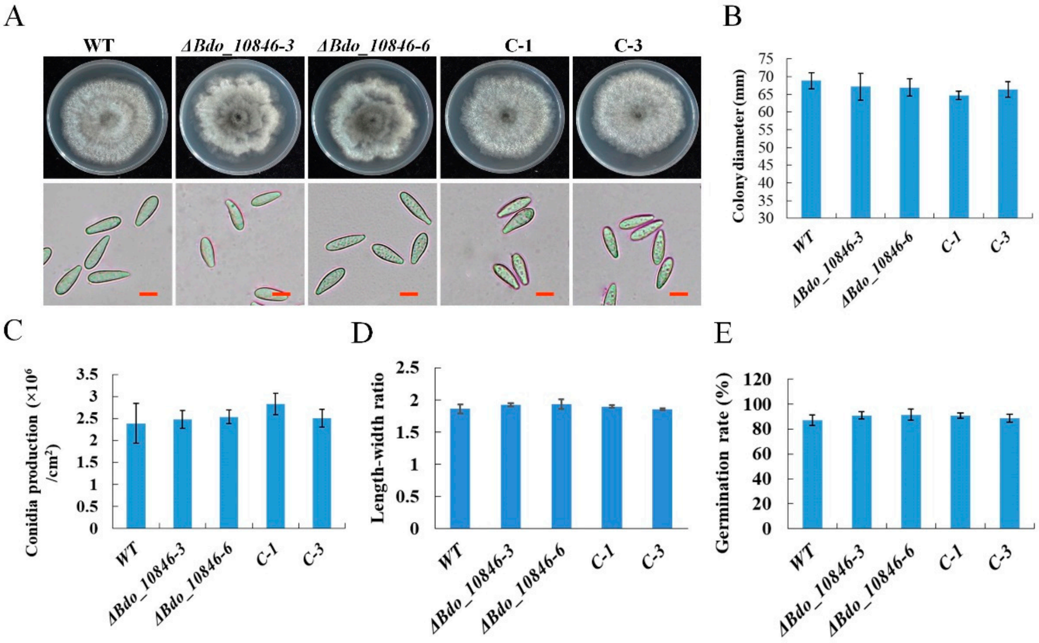

Figure S4. Mycelial growth and sporulation of *Bdo_10846* knockout transformants and WT strains. (**A**) Colonies and conidia morphology of WT, two *Bdo_10846* knockout transformants, and two complementary transformants, Bar = 10 μm. (**B–E**) Statistical results of colony diameters, conidial production, conidial length-width ratio, and conidial germination rate. Data were averages (and standard errors) of values from two independent experiments. Statistical significance was analyzed with one-way analysis of variance (ANOVA) and Duncan´s test, no significant difference was detected ($p < 0.05$).

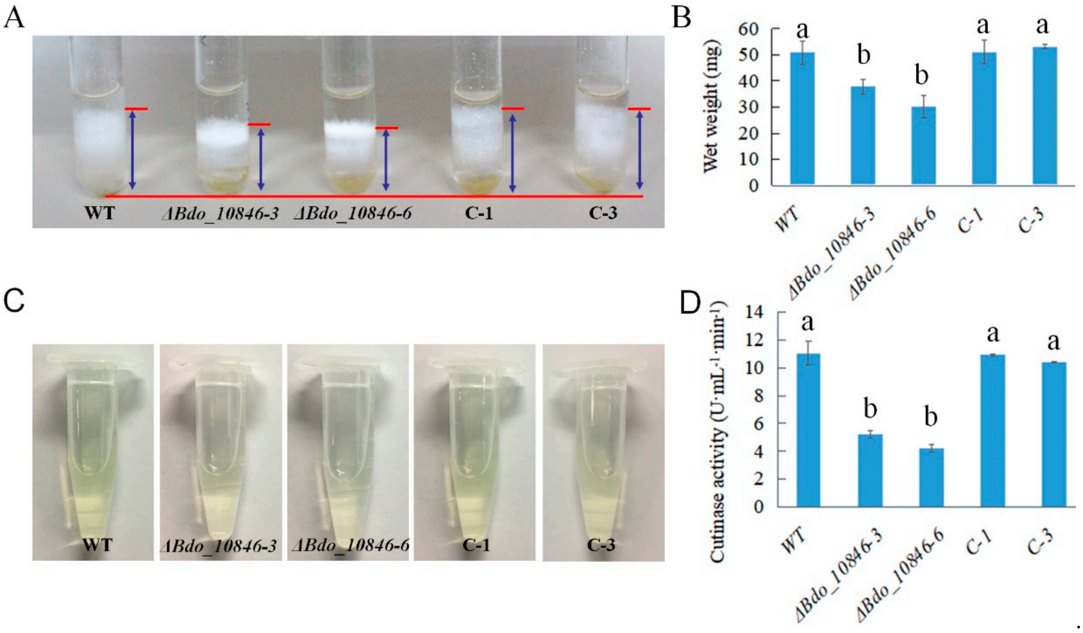

Figure S5. The mycelial growth and the cutinase activities of *Bdo_10846* knockout transformants in medium with cutin as carbon source. (**A**) Growth of WT and transformants (two *Bdo_10846* knockout transformants *ΔBdo_10846-3*, *ΔBdo_10846-6* and two complementary transformants C-1, C-3) in cutin broth for 10 days. (**B**) Mycelium wet weight. (**C**–**D**) Cutinase activity in medium supernatant assayed using ϱ-nitrophenyl butyrate. Yellow color depth represented the cutinase activity. Data were averages (and standard errors) of two independent experiments. Statistical significance was analyzed with one-way analysis of variance (ANOVA) and Duncan´s test, and different letters indicate significant difference ($p < 0.05$).

Table S1. Information of the predicted 13 cutinases in *Botryosphaeria dothidea.*

Gene ID	SP	Cleavage Site	Protein Length	Conserved Domain				
				From	To	E-Value	Bit Score	Predicted Function
Bdo_01641	Y	17 and 18	228	40	225	9.07E-44	144	Cutinase
Bdo_01702	Y	18 and 19	220	38	206	2.70E-45	148	Cutinase
Bdo_03157	Y	19 and 20	236	29	234	1.69E-32	115	Cutinase
Bdo_03766	Y	18 and 19	323	52	225	2.50E-52	169	Cutinase
Bdo_04428	Y	19 and 20	208	37	205	3.28E-48	155	Cutinase/Esterase
Bdo_04657	Y	16 and 17	606	418	603	1.73E-45	158	Cutinase
Bdo_04821	Y	17 and 18	234	35	226	1.37E-33	118	Cutinase
Bdo_05778	Y	19 and 20	225	28	217	3.58E-34	120	Cutinase
Bdo_08566	Y	19 and 20	212	38	206	2.17E-46	150	Cutinase/Esterase
Bdo_10693	Y	18 and 19	283	54	189	5.42E-43	144	Cutinase
Bdo_10846	Y	18 and 19	228	53	223	1.24E-55	174	Cutinase
Bdo_12612	Y	18 and 19	234	56	231	4.15E-54	171	Cutinase
Bdo_13750	Y	18 and 19	239	39	236	6.77E-47	153	Cutinase

Table S2. Information of genes involved in suberin synthesis process.

Organism	ID	Gene	Protein	Function
Malus domestica	*mdp0000233110*	*MdLFAD*	Long-chain fatty alcohol dehydrogenase family	Long-chain fatty acid metabolic process (Legay et al., 2015)
	mdp0000228252	*MdMYB93*	MYB Transcription factor	Regulator of the suberin synthesis (Legay et al., 2016)
	mdp0000312405	*MdFCoAT*	Feruloyl-CoA acyl-transferase family protein	Regulating the peridermal water permeability (Legay et al., 2016)
Nicotiana benthamiana	*Nbv5tr6203422*	*NbFAR3*	Fatty acyl reductases	Fatty-acyl-CoA reductase (alcohol-forming) activity (Kosma et al., 2014)
	Nbv5tr6232718	*NbGPAT*	Glycerol phosphate acyl transferase	Glycerol-3-phosphate O-acyltransferase activity (Beisson et al., 2007)
	Nbv5tr6216569	*NbGDSL*	GDSL-esterase	O-acyltransferase activity ǀ lipase activity (Girard et al., 2012)

Table S3. Primers used for qRT-PCR.

Gene	Primers	Sequence
MdFCoAT	YG 405-F	TGGTGACGAGCAGTTACATGAGGTC
	YG 405-R	CAGCCAAAGTCAGTTGTGTGGAAACT
MdLFAD	YG 110-F	GCTGTGGACAATTTACTCATCTGCTCA
	YG 110-R	TGGATTCACACCAACTGCAGTCGG
MdMYB93	YG 252-F	GCTGGATATTCAAGACCCTACTGCAC
	YG 252-R	TCTGGAGCTTGATCATTAACCATGTCC
MdGAPDH	GAPDH-F	GCAAAGAAGGTTATCATCTCTGCCCC
	GAPDH-R	GGTGCAACTAGCATTGGAAAGAATGTGG
MdNPR1	MdNPR1-F	CATTGCCCATGCGGAGACATCTGA
	MdNPR1-R	GAGCAATGAGGGAAGTAGCATCTACC
MdPR1	MdPR1-F	GGCGACTGCAATCTCGTGCACT
	MdPR1-R	GACTCATAACTGTAGTCGGCTTTCTC
MdPDF1.2	MdPDF1.2-F	AGCCACGACAATTGCCTCCAGG
	MdPDF1.2-R	GATGATGGGACCACTGCTTGCG
Bdo_10846	YG 10846-F	TCGTCGGAGGTGGATATAGTCAAG
	YG 10846-R	TTCAGCTTCTCCTGCGGAAGAC
Bdo_Actin	YG ACTIN-F	CAACTGGGACGACATGGAGAAGATTTG
	YG ACTIN-R	GATCTGGGTCATCTTCTCACGGTTG
NbEF1-α	NbEF-F	CTGCCAGCTTTACCTCCCAAGTCA
	NBEF-R	CCAGAACGCCTGTCGATCTTGGT
NbPR1	NbPR1-F	GACGACCAGGTAGCAGCCTATG
	NbPR1-R	CAACAGCCTTAGCAGCCGTCATG
NbNPR1	NbNPR1-F	TGCAGCAGACGATGTAATGGTGGT
	NbNPR1-R	CTTGTAGACCAAGTTCTGCTCGTG
NbPDF1.2	NbPDF1.2-F	GTTACTTCTAGCATTGCTTGTCATGGC
	NbPDF1.2-R	CGGTGGCACAGTTGCTATCTCTTG
NbFAR3	NbFAR3-F	TACCTTCAACCCAACCAGGA
	NbFAR3-R	TTATGGATGGCCAAACACCT
NbGPAT	NbGPAT-F	TCATGCAACAACAGCTAGGG
	NbGPAT-R	ACGTGGCTTCTACTGGCAAC
NbGDSL	NbGDSL-F	TTTGCACATGTTCCTGGAGA
	NbGDSL-R	TTGTCACTCACCCAGCTGAT

Table S4. Primers used for plasmid construction and transformants verification.

Primer Name	Primer Sequence (5′–3′)
	Gene Knockout Plasmid Construction
10846up-F	CTTGCAAGTCACATGGCTTACAAAGT
10846up-R	ACCGGTCACTGTACAGAGCTCACGTTCGAATTGAGAATGACGGGGAAAGAT
10846down-F	GGATATAAGATCGTTGGTGTCGGTCAATACGCGACTTTCGTTTGCT
10846down-R	CTGAAAGACGGTGAATATGCAGTGT
10846nest-F	GGAAGACACACCAAAGACGTTGAACTC
10846nest-R	CGAAAGGAGCTTGGCGCATTCATC
Hyg-F	CGTGAGCTCTGTACAGTGACCGGT
Hyg-R	CGACACCAACGATCTTATATCCAGATTCG
	Complementary Plasmid Construction
10846HB-F	GACCTGCAGGCATGCAAGCTTTGCATGGATACCGTTTCAGATTACTGC
10846HB-R	GACCATGATTACGCCAAGCTTTCTGACTCGCCTTCAACCAACAAG
	PCR Verification of Gene Knockout Transformants
P1	TATAGCTGCGGAGTAAAGATTCCAAGT
P2	AATCATCCACTGCACCTCAGAGC
P3	ACCGCGGGATCCACTTAACGTTAC
P4	AATCATCCACTGCACCTCAGAGC
P5	GTCTCCGTGCTCTTTGCTAGCTT
P6	TGCGATCAGCCAGGAAGGACGA
	Probe Labeling
HYG Probe-F	CTCCCGATTCCGGAAGTGCTTGA
HYG Probe-R	CAACCACGGCCTCCAGAAGAAGATGTT
	Bdo_10846 Overexpressing
GFP-F	ATCGATACCGTCGACCTCGAGATGGTGAGCAAGGGCGAGGAG
GFP-R	GGCCTTAGCATGCGAAGATCTTTACTTGTACAGCTCGTCCATGCC
H3P-F	AAGCGCGTTGGATTAGAGGTCGACAGGGCAAGCGCAAGATCATTTGTC
H3P-R	CTTGCTCACCATCTCGAGGTCGACaagcttGCAGAAGTTGTGTTGGGTCGGAAA
10846OE-F	CACAACTTCTGCAAGaagcttATGAGGGTCACTGCGACTATCG
10846OE-R	CATCTCGAGGTCGACaagcttCTGAGCCGCCCTGATGCGATC
	Prokaryotic Expression of Bdo_10846
pHAT10846-F	CACCATCACCATCACTCCATGTCTCCCATCAATGTTGAAGTCCGC
pHAT10846-R	AGAGGGCCCGGATCCCTCGAGCTACTGAGCCGCCCTGATGCG
